# Genome-Wide Dissection of the Genetic Basis for Drought Tolerance in *Gossypium hirsutum* L. Races

**DOI:** 10.3389/fpls.2022.876095

**Published:** 2022-06-28

**Authors:** Xinlei Guo, Yuanyuan Wang, Yuqing Hou, Zhongli Zhou, Runrun Sun, Tengfei Qin, Kunbo Wang, Fang Liu, Yuhong Wang, Zhongwen Huang, Yanchao Xu, Xiaoyan Cai

**Affiliations:** ^1^Henan Institute of Science and Technology, Collaborative Innovation Center of Modern Biological Breeding of Henan Province, Henan Key Laboratory Molecular Ecology and Germplasm Innovation of Cotton and Wheat, Xinxiang, China; ^2^Institute of Vegetables and Flowers, Chinese Academy of Agricultural Sciences, Beijing, China; ^3^State Key Laboratory of Cotton Biology, Institute of Cotton Research, Chinese Academy of Agricultural Sciences, Anyang, China

**Keywords:** cotton, *G. hirsutum* race, drought, genome-wide association study, Cotton80KSNP, GAPIT, candidate gene prediction

## Abstract

Drought seriously threats the growth and development of *Gossypium hirsutum* L. To dissect the genetic basis for drought tolerance in the *G. hirsutum* L. germplasm, a population, consisting of 188 accessions of *G. hirsutum* races and a cultivar (TM-1), was genotyped using the Cotton80KSNP biochip, and 51,268 high-quality single-nucleotide polymorphisms (SNPs) were obtained. Based on the phenotypic data of eight drought relative traits from four environments, we carried out association mapping with five models using GAPIT software. In total, thirty-six SNPs were detected significantly associated at least in two environments or two models. Among these SNPs, 8 and 28 (including 24 SNPs in 5 peak regions) were distributed in the A and D subgenome, respectively; eight SNPs were found to be distributed within separate genes. An SNP, TM73079, located on chromosome D10, was simultaneously associated with leaf fresh weight, leaf wilted weight, and leaf dry weight. Another nine SNPs, TM47696, TM33865, TM40383, TM10267, TM59672, TM59675, TM59677, TM72359, and TM72361, on chromosomes A13, A10, A12, A5, D6, and D9, were localized within or near previously reported quantitative trait loci for drought tolerance. Moreover, 520 genes located 200 kb up- and down-stream of 36 SNPs were obtained and analyzed based on gene annotation and transcriptome sequencing. The results showed that three candidate genes, *Gh_D08G2462, Gh_A03G0043*, and *Gh_A12G0369*, may play important roles in drought tolerance. The current GWAS represents the first investigation into mapping QTL for drought tolerance in *G. hirsutum* races and provides important information for improving cotton cultivars.

## Introduction

Cotton is the most important fiber crop and an important oilseed crop. The genus *Gossypium* contains more than 50 species, of which four are cultivated. *G. hirsutum* L. (AD_1_), also known as Upland cotton, is the most widely cultivated cotton plant worldwide; it is grown on approximately 95% of cotton acreage and accounts for more than 95% of cotton production because of its high yield potential, attractive fiber quality, and wide adaptability. Even though *G. hirsutum* L. is adapted to subtropical climates where drought is prevalent, it is not recognized as a drought-tolerant crop and is not very efficient in terms of water use (Dabbert and Gore, 2014; Abdelraheem et al., 2019). Drought has a deleterious effect on cotton at all growth stages and overall, productivity and quality are seriously diminished. Moreover, with the increasing global population, the increasing water demand for agriculture, and the decreasing availability of fresh water, the impact of drought will be further exacerbated (Gupta et al., 2020). Therefore, determining the mechanisms underlying the response of *G. hirsutum* to drought stress and making full use of natural genetic variation for improving drought tolerance (DT) in cotton cultivars are urgent needs for cotton breeding programs.

Drought relative traits are complex quantitative traits that are controlled by quantitative trait loci (QTL) and environmental factors. Moreover, there is a complicated relationship between drought-tolerance, quality, and yield. Therefore, it is difficult to improve these three traits simultaneously using traditional breeding techniques. Using molecular marker techniques to identify QTL for drought relative traits and applying the molecular markers that are closely linked to, or significantly associated with, target QTL for marker-assisted selection (MAS) could rapidly produce drought-tolerant cotton varieties with high yield and excellent quality. Linkage mapping is a traditional method for gene/QTL analysis. To dissect the genetic variation underlying drought relative traits, ample QTL have been identified based on intra- and interspecific genetic linkage maps (Saeed et al., 2011; Abdelraheem et al., 2015a,b; Amjid et al., 2015; Pauli et al., 2016; Zheng et al., 2016; Pawlowicz et al., 2017; Ulloa et al., 2020). These QTL provide ample marker resources for MAS in drought-tolerant cotton breeding programs.

However, limited recombination events and low genetic diversity in the limited parental mapping population are major obstacles for conventional linkage mapping. Genome-wide association study (GWAS), as an alternative strategy, has been found to be an effective method with unique advantages for identifying the genetic variants controlling traits. Several researchers have reported markers associated with drought tolerance in *G. hirsutum* L. (Jia et al., 2014; Hou et al., 2018; Li B. Q. et al., 2020; Abdelraheem et al., 2021b). Jia et al. (2014) performed association mapping for DT based on 323 *G. hirsutum* accessions, and identified 15 significantly associated markers. Hou et al. (2018) performed association mapping with 319 Upland cotton accessions and detected 20 quantitative trait nucleotides (QTNs) significantly associated with 6 drought tolerance-related traits. Li B. Q. et al. (2020) used a natural population of 517 Upland cotton accessions to conduct association mapping and discovered 39 QTL related to drought tolerance. Abdelraheem et al. (2021a) carried out association mapping for DT and identified 53 QTL based on an association mapping panel of 376 Upland cotton accessions. Using a multiparent advanced generation intercross population of 550 recombinant inbred lines (RILs) together with their 11 Upland cotton parents, Abdelraheem et al. (2021b) carried out association mapping for drought tolerance at the seedling stage and detected 20 QTL including 13 and 7 QTL for plant height and dry shoot weight (DSW), respectively. These studies have supplemented lots of marker information for molecular marker-assisted breeding.

Although the studies above have been performed to improve the drought tolerance of cotton, the accessions they used were cultivars which were domesticated and selected repeatedly for their high yield traits. The genetic base of cotton is so narrow that it is necessary to broaden our germplasm resources for a high level of genetic diversity. *G. hirsutum* races, named semi-wild cotton, are the progenitors of cultivated *G. hirsutum* and are classified into seven races according to their geographical distribution. *G. hirsutum* races have wide phenotypic and high genetic diversity levels (Liu et al., 2000; Lacape et al., 2006; Abdurakhmonov et al., 2008; Hinze et al., 2015; Zhang et al., 2019). Moreover, there is no reproductive isolation between *G. hirsutum* races and cultivars. Many researchers have used *G. hirsutum* races to broaden the genetic base for improving yield (Zhang et al., 2016; Liu X. et al., 2021), fiber quality (Feng et al., 2019, 2020), cotton fiber color (Liu D. et al., 2021; Liu X. et al., 2021), and abiotic and/or biotic stress tolerance (Hinze et al., 2015; Wang et al., 2016; Xu et al., 2020). In this study, we used a natural population consisting of 188 accessions of *G. hirsutum* races and TM-1 to carry out a GWAS of drought tolerance based on the phenotypes in 4 different environments and the genotypes performed by the Cotton80KSNP biochip. This study aims to uncover the genetic variation in drought tolerance, to select candidate genes, and to predict the breeding potential of lines for the genetic improvement of drought-tolerance traits.

## Materials and Methods

### Plant Materials and Growth Conditions

The association mapping panel consisted of 189 accessions (Supplementary Table 1), which represented all seven races according to geographical distribution. Among them, 188 accessions were selected from *G. hirsutum* races (including 20 punctatum, 113 latifolium, 18 marie-galante, 7 palmeri, 20 morrilli, 2 yucatanense and 8 richmondi). A representative of cultivated Upland cotton, the genetic standard *G. hirsutum* L. acc. Texas Marker-1 (TM-1), was drought-tolerant, and was used as the control. All the accessions were perennially preserved and purified in the National Wild Cotton Nursery, Sanya, Hainan, China, which is supervised by the Institute of Cotton Research of Chinese Academy of Agricultural Sciences (ICR-CAAS), Anyang, Henan, China. The 188 accessions of *G. hirsutum* races were originally introduced from the USDA-ARS Southern Agricultural Research Center in College Station, Texas, USA. All the accessions were legally planted in the field/greenhouse with a single row plot during 2015–2016 and 2016–2017 in Damao, Yacheng, and Baogang, Hainan, China. In each environment, 20–25 plants were arranged in each row, with a row length of 5.0 m and a row interval of 1.0 m. Normal watering experiments were performed in the field, and drought stress experiments were carried out in the greenhouse. For all the other activities, standard local management practices were performed. For description purposes, the four environments, 2015–2016 Damao in the field, 2015–2016 Damao in the greenhouse, 2016–2017 Yacheng in the greenhouse, and 2016–2017 Baogang in the field, were designated E_1_, E_2_, E_3_, and E_4_, respectively.

### Evaluation of Drought-Tolerance Traits

Eight traits contributing to drought tolerance, i.e., chlorophyll content (CHL, unit; measured using a SPAD 502 Plus (Minolta Instruments, Osaka, Japan)), leaf area (LA, cm^2^), plant height (PH, cm), stem diameter (SD, cm), number of leaves (LEAVES), leaf fresh weight (LFW, g plant^−1^), leaf wilted weight (LWW, g plant^−1^), and leaf dry weight (LDW, g plant^−1^), were investigated (Supplementary Table 2). Ten consecutive plants in the middle of each row were tagged for trait phenotyping. To reduce environmental error, we estimated the best linear unbiased prediction (BLUP) for each trait per genotype using the lme4 package in the R program. Both the BLUP and single environment values were imported for association mapping. We used R to carry out the descriptive statistical analyses, to draw the histograms for the phenotypic change trends in each environment, to perform ANOVA, and to realize the correlation analysis.

### DNA Extraction and SNP Genotyping

The seeds of each accession were sown in soil with added nutrients in the greenhouse, and young leaves of each individual were collected at the seedling stage. High-quality genomic DNA was extracted by the modified CTAB method, and the concentration was diluted to 50 ng/μL. The genomic DNAs of all samples were hybridized to the CottonSNP80K biochip according to the Illumina protocols (Illumina Inc., San Diego, USA). The specifically hybridized loci were processed for single base extension, stained, and imaged on an Illumina iScan Reader. GenomeStudio Genotyping software (v1.9.4, Illumina) was used to cluster the row data for SNP genotype calls (Cai et al., 2017). The raw genotyping data were filtered, and polymorphic SNPs with a minor allele frequency (MAF) <0.05, integrity < 50%, or call rate <90% were removed.

### Population Structure and Kinship Analysis

Only SNPs with a MAF ≥ 0.05, integrity ≥ 50%, and call rate ≥ 90% were used for population structure and kinship analyses. Population structure was determined using ADMIXTURE software (Alexander et al., 2009). The number of genetic clusters (K) was predefined as 1–20 to explore the population structure. This analysis provided cross-validation (CV) error estimates derived from each of the K populations, and the K with the minimum number of CV errors was taken as the number of subgroups. The kinship coefficient of each pair of individuals was calculated using Tassel 5.0 software. Linkage disequilibrium (LD) analysis was performed to determine the mapping resolution for GWAS. Pairwise LD between markers was calculated as the squared correlation coefficient (R^2^) of alleles using LDdecay software.

### GWAS and Candidate Genes Selection

The GWAS was performed using the general linear model (GLM), mixed linear model (MLM), compressed linear mixed model (CMLM), settlement of kinship under progressively exclusive relationship (SUPER), and fixed and random model circuitous probability unification (FarmCPU), provided by the genome associated prediction integrated tool (GAPIT) program. For all of the models, the “PCA.total” was set as two. Manhattan plots were drawn using the R package qqman (Turner, 2014). The significance levels of the associations between SNPs and traits were estimated based on the threshold of the Bonferroni correction for multiple tests (1/n), where n was the total number of SNPs used in the association mapping.

To identify potential candidate genes for drought tolerance, we collected genes located within 400 kb (200 kb upstream and downstream) of significantly trait-associated SNPs based on TM-1 genome sequencing data (NBI v1.1) (Zhang et al., 2015). Then, we implemented the gene annotations from Gene Ontology (GO) analysis (http://www.geneontology.org/) (Ashburner et al., 2000).

### Expression Patterns Analysis of Candidate Genes

To analyze the expression patterns of candidate genes, *G. hirsutum* race marie-galante85 (Mar85) and *G. hirsutum* race latifolium40 (Lat40) were used. The seeds of the two accessions were germinated in filter paper rolls at 28°C under 16 h light/8 h dark conditions. The seedlings were grown in Hoagland nutrient solution for 3 weeks and then transferred to 17% polyethylene glycol 6000 (PEG6000) solution. The root and leaf samples were collected at 0, 24, and 48 h after treatment. The sampling was carried out with three repeats. High-quality total RNA from each sample was extracted using TRIzol Reagent (Life Technologies, California, USA) following the manufacturer's instructions. After constructing the cDNA libraries, performing sequencing and quality control, mapping the reads to the TM-1 genome (Zhang et al., 2015), and quantifying, we calculated the fragments per kilobase of transcript per million fragments mapped reads (FPKM) values. For each accession, the normalized values represented the expression level of each candidate gene, and heatmaps of the candidate genes expression patterns were created using Mev 4.9 (Saeed et al., 2003).

## Results

### Phenotypic Variation and Correlation Analysis

We evaluated eight drought-tolerance traits of 189 accessions across four environments during 2015–2016 and 2016–2017 (Table 1). All eight traits displayed broad variation when grown under individual environmental condition. The average phenotypic values of the traits were as follows: CHL, 50.18 (range 35.43–66.72); LA, 130.68 cm^2^ (range 23.70–249.64 cm^2^); PH, 69.34 cm (range 26.20–146.10 cm); SD, 0.95 cm (range 0.37−2.12 cm); LEAVES, 15.115 (range 5.1–31.6); LFW, 16.02 g plant^−1^ (range 1.99–43.74 g); LWW, 11.35 g plant^−1^ (range 0.82–50.83 g); and LDW, 3.78 g plant^−1^ (range 0.28–12.58 g). For the eight traits, the coefficient of variation (CV) ranged from 6.87% (CHL in E_2_) to 43.17% (LDW in E_3_). As indicated by the descriptive statistics, the traits exhibited an approximately normal distribution in all environments, and the histogram of the traits investigated in each environment showed that drought-tolerance traits exhibited the genetic characteristics of quantitative traits, with continuous distributions across different environments (Supplementary Figure 1).

**Table 1:** Descriptive statistics for the eight drought-tolerance related traits across four environments.

**Traits**	**Env**.	**Mean**	**Max**	**Min**	**Std**	**CV (%)**	***H^2^***
CHL	E_1_	55.46	66.72	42.28	4.03	7.27	0.3771
	E_2_	48.11	56.86	40.57	3.29	6.87	
	E_3_	49.27	60.06	38.13	3.91	7.94	
	E_4_	47.86	57.93	35.43	3.9	8.16	
LA (cm^2^)	E_3_	124.82	210.48	34.36	28.03	22.46	0.599
	E_4_	136.54	249.64	23.7	32.88	24.08	
PH (cm)	E_1_	77.88	146.1	40.58	16.14	20.68	0.3061
	E_2_	47.32	139	26.2	12.93	27.33	
	E_3_	67.69	103.37	35.23	12.43	18.36	
	E_4_	84.45	124.3	54.7	11.44	13.55	
SD (cm)	E_1_	1.25	2.12	0.77	0.23	18.08	0.3364
	E_2_	0.54	1.4	0.37	0.1	18.48	
	E_3_	0.87	1.24	0.55	0.132	15.19	
	E_4_	1.14	1.87	0.71	0.144	12.64	
LEAVES	E_1_	20.68	31.6	15.03	3.11	15.03	0.5901
	E_2_	9.55	24.5	5.1	2.55	26.69	
LFW (g plant^−1^)	E_1_	13.93	27.13	2.01	4.45	31.92	0.5813
	E_2_	8.18	15.4	1.99	2.46	30.08	
	E_3_	19.44	38.39	2.26	6.15	31.65	
	E_4_	22.52	43.74	3.55	6.67	29.62	
LWW (g plant^−1^)	E_1_	9.18	21.22	0.61	3.67	40.03	0.5418
	E_2_	6.58	12.51	1.19	2.05	31.18	
	E_3_	14.31	50.83	0.82	5.33	37.27	
	E_4_	15.33	30.06	1.71	5.07	33.06	
LDW (g plant^−1^)	E_1_	3.29	6.38	0.44	1.06	32.23	0.5066
	E_2_	1.98	3.82	0.28	0.6	30.05	
	E_3_	4.93	12.58	0.42	2.13	43.17	
	E_4_	4.91	11.43	0.65	1.57	31.99	

*E_1_, E_2_, E_3_, and E_4_ indicate the four environments 2015–2016 Damao in the field, 2015-2016 Damao in the greenhouse, 2016-2017 Yacheng in the greenhouse, and 2016-2017 Baogang in the field, respectively; CHL, chlorophyll content; LA, leaf area; PH: plant height; SD, stem diameter; LEAVES, number of leaves; LFW, leaf fresh weight; LWW, leaf wilted weight; LDW, leaf dry weight; and CV, coefficient of variation*.

**Figure 1:**
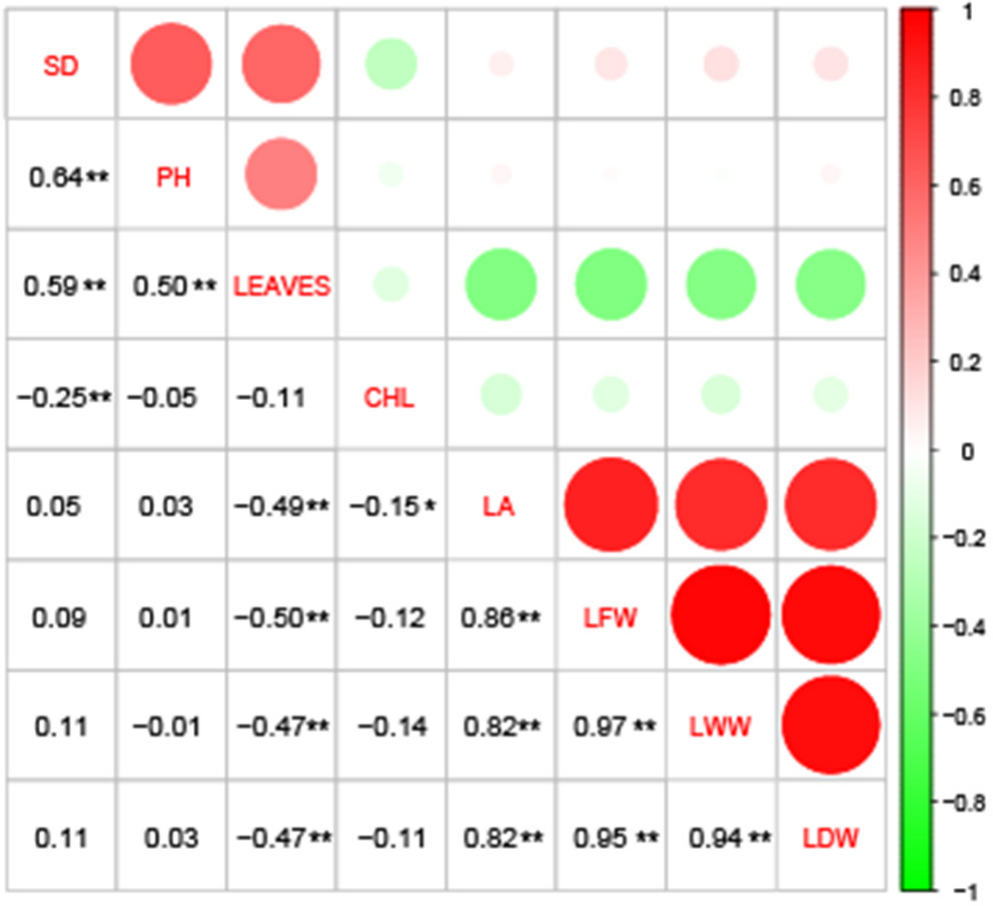
Correlation analysis among the eight drought-tolerance traits of *Gossypium hirsutum* races. CHL, chlorophyll content; LA, leaf area; PH, plant height; SD, stem diameter; LEAVES, number of leaves; LFW, leaf fresh weight; LWW, leaf wilted weight; and LDW, leaf dry weight. *, significant at the α = 0.05 level; **, significant at the α = 0.01 level.

The correlation analysis showed that LA, LFW, LWW and LDW were significantly, highly, and positively correlated, with a correlation coefficient between 0.82 and 0.97; LEAVES showed a significant and moderate negative correlation with those four traits, with a correlation coefficient between 0.47 and 0.50, but showed a significant and moderate positive correlation with SD and PH, with a correlation coefficient between 0.50 and 0.59. Furthermore, CHL and LA, CHL and SD showed weakly negative correlations (Figure 1). The broad-sense heritability (*H*^2^) of the eight traits were estimated based on ANOVA. The *H*^2^ for PH (0.3061), SD (0.3364) and CHL (0.3771) were low (<0.5), and the *H*^2^ for LDW (0.5066), LWW (0.5418), LFW (0.5813), LEAVES (0.5901), and LA (0.5990) were moderate (> 0.5 but < 0.7) (Abdelraheem et al., 2021a). The results indicate that 30–60% of the phenotypic variation of these traits was determined by genetic factors.

### Distribution of Polymorphic SNPs

**Figure 2:**
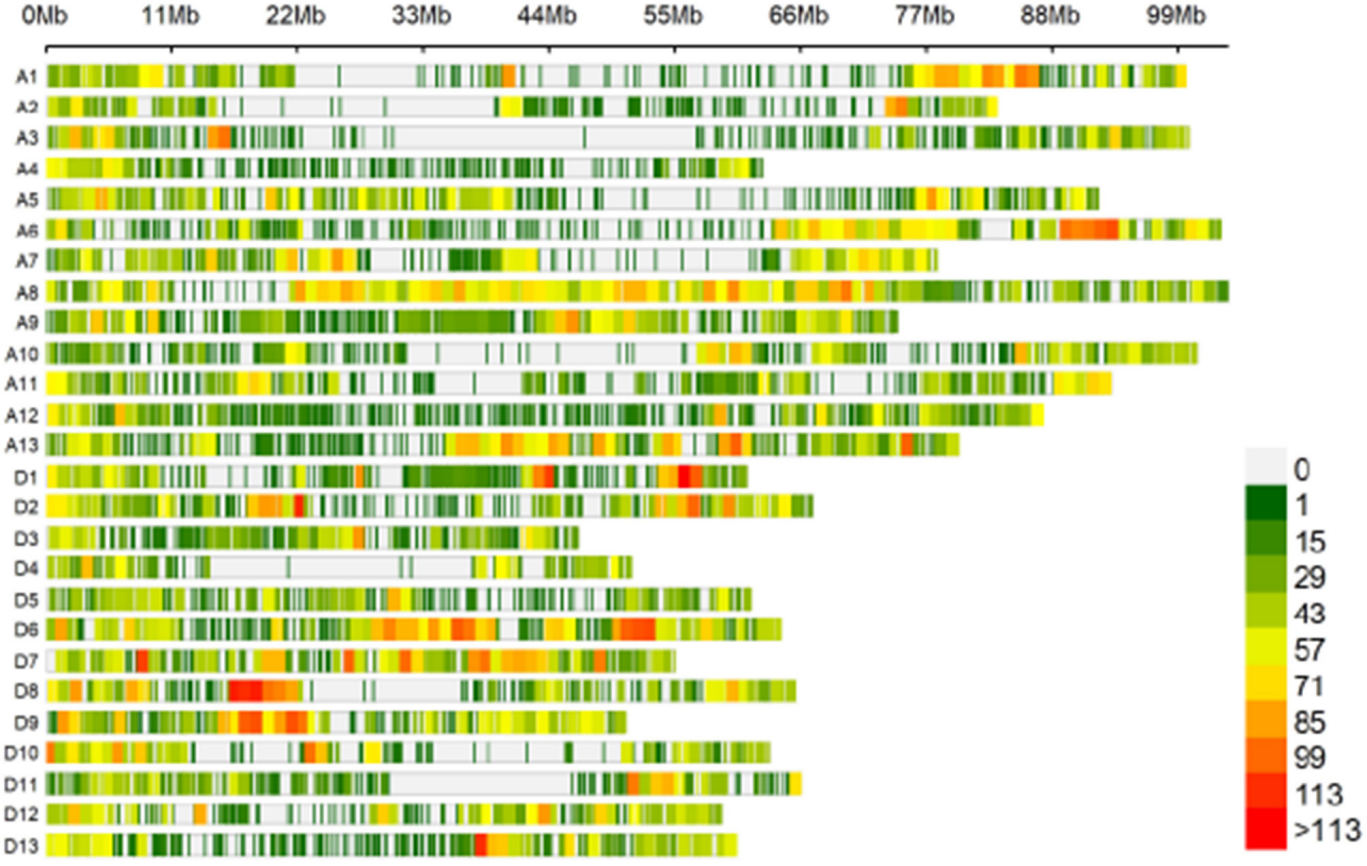
Single-nucleotide polymorphism (SNP) distributions on the 26 chromosomes of *G. hirsutum* races. A1–A13 and D1–D13 on the vertical axis represent the serial numbers of the 26 chromosomes; the horizontal axis shows chromosome length; the window size is 1 Mb.

The 189 accessions were genotyped by CotttonSNP80K arrays, with the Illumina GenomeStudio software. In total, 51,268 filtered SNPs were detected based on a MAF ≥ 0.05, integrity ≥ 50%, and call rate ≥ 90%, and they were unevenly distributed throughout the *G. hirsutum* genome (Table 2; Figure 2), with 27,673 (54%) and 23,596 (46%) SNPs in the A and D subgenomes, respectively. The average marker density was approximately one SNP per 38.66 kb. In A subgenome, chromosome A8 contained the largest number of markers (4,644), with a marker density of one SNP per 22.26 kb, and chromosome A4 had the fewest markers (918), with a marker density of one SNP per 68.32 kb. For the D subgenome, chromosome D6 had the most markers (2,954), with a marker density of one SNP per 21.76 kb, and D4 had the fewest markers (939), with a marker density of one SNP per 54.63 kb. The polymorphism information content (PIC) values ranged from 0.011 (TM50634) to 0.597 (TM29059) among the SNPs, and from 0.295 (chromosome D8) to 0.341 (chromosome A8) among the chromosomes. The mean PIC values of the A and D subgenome were 0.3271 and 0.3275, respectively, and the mean PIC value of the whole genome was 0.3273.

**Table 2:** Summary of SNPs in the 26 chromosomes of *G. hirsutum* races.

**Chr**.	**Size (kb)**	**No. SNPs**	**SNP density (kb/SNP)**	**PIC**	**Chr**.	**Size (kb)**	**No. SNPs**	**SNP density (kb/SNP)**	**PIC**
A1	99,872.14	2,154	46.37	0.315	D1	61449.80	1,687	36.43	0.337
A2	83,336.17	1,205	69.16	0.314	D2	67184.59	2,182	30.79	0.328
A3	100,110.37	1,441	69.47	0.327	D3	46,626.67	1,246	37.42	0.315
A4	62,720.68	918	68.32	0.315	D4	51,298.02	939	54.63	0.332
A5	92,046.42	2,303	39.97	0.325	D5	61,813.40	1,443	42.84	0.324
A6	102,823.40	2,731	37.65	0.325	D6	64,293.64	2,954	21.76	0.338
A7	78,085.31	1,977	39.50	0.329	D7	55,005.83	2,516	21.86	0.333
A8	103,394.49	4,644	22.26	0.341	D8	65,542.20	2,110	31.06	0.295
A9	74,545.06	2,199	33.90	0.328	D9	50,864.12	2,227	22.84	0.336
A10	100,812.92	1,837	54.88	0.328	D10	63,359.40	1,571	40.33	0.332
A11	93,148.00	1,968	47.33	0.327	D11	66,015.52	1,399	47.19	0.320
A12	87,232.29	1,660	52.55	0.313	D12	59,104.84	1,752	33.74	0.331
A13	799,13.54	2,636	30.32	0.333	D13	60,365.11	1,570	38.45	0.328

*PIC, polymorphism information content*.

### Population Structure and Relative Kinship Analysis

**Figure 3:**
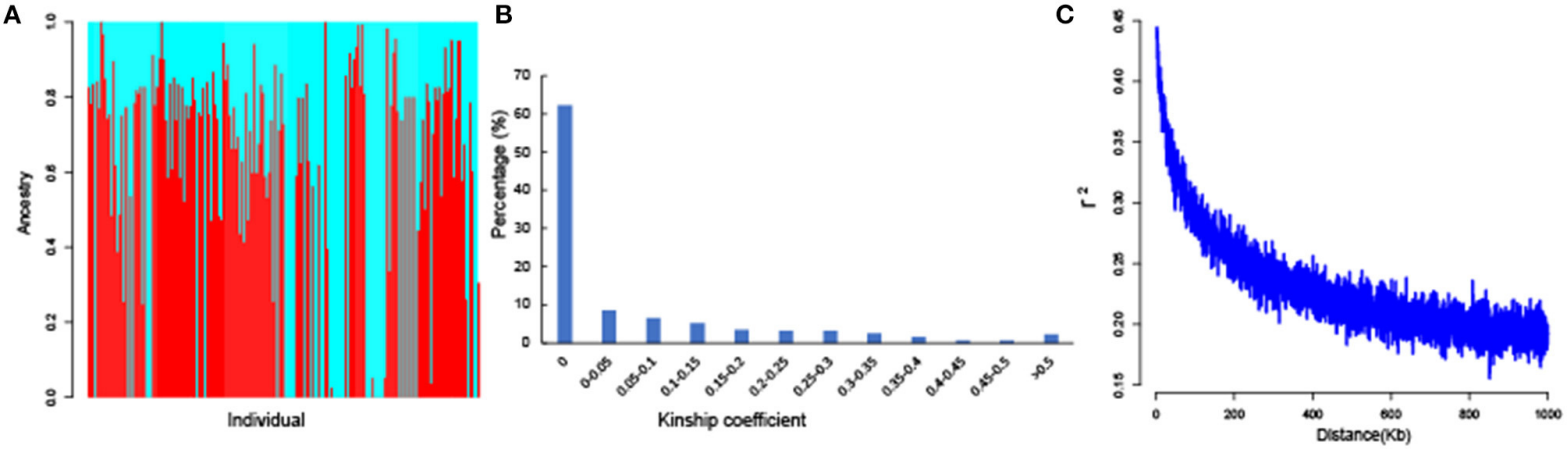
The results of population structure, kinship coefficients, and linkage disequilibrium (LD) decay of 189 *G. hirsutum* accessions. **(A)** The total panel was divided into two subgroups, and each color represents one subgroup. **(B)** Histogram of the frequency distribution of pairwise relative kinship coefficients. **(C)** Genome-wide average LD decay estimated for the whole genome.

Population structure analysis of the 189 accessions was performed by ADMIXTURE with all 51,268 SNPs, and the results indicated that CV error was minimal at K = 2. Therefore, the optimum K was 2, and the tested population could be separated into two subgroups (Figure 3A). Subgroups 1 and 2 included 90 and 99 accessions, respectively. Most of the accessions in each subgroup had mixed ancestry, and an obvious geographic subpopulation structure was not observed, indicating that these accessions might have experienced introgression or gene flow in the complex natural environment. Moreover, 51,268 high-quality SNPs were used to evaluate the kinship coefficient. The kinship coefficient of each pair of individuals was calculated using Tassel software. A pairwise relative kinship value of 0 accounted for 62.31% of all the kinship coefficients. In addition, kinship values from 0 to 0.05 accounted for more than 70.91% of all the pairwise relative kinship coefficients (Figure 3B). Only 2.12% of the pairwise relative kinship coefficients were > 0.5. The kinship analysis indicated that most accessions had no, or a weak, relationship with each other in the population. Moreover, LD decay was measured using all 51,268 SNPs. The LD analysis estimated the average LD decay distance of our population across the whole genome to be approximately 550 kb, with an r^2^ = 0.5 (Figure 3C). These results indicated that the association population was suitable for association mapping.

### Genome-Wide Association Mapping

Genome-wide association mapping for the eight drought traits was performed using the GLM, MLM, CMLM, SUPER, and FarmCPU models, which were provided by the GAPIT program. SNPs with -log_10_*P* values >4.71 (1/51, 268) were selected as significantly trait-associated SNPs (Sun Z. et al., 2017). Here, based on the 5 models, 30, 43, 26, 34, and 190 SNPs randomly distributed on 26 chromosomes were associated with the 8 drought-tolerant traits based on the values for each environment, including BLUPs (Supplementary Table 3). For CHL, 12, 16, 16, 16, and 18 SNPs were identified using the GLM, MLM, CMLM, SUPER, and FarmCPU models, respectively; 4, 3, 1, 3, and 28 were associated with PH; 6, 5, 1, 3, and 14 were associated with SD; 1, 12, 3, 4, and 25 were associated with LWW; and 3, 4, 3, 4, and 41 were associated with LDW. For LA, 1, 1, and 21 SNPs were identified by GLM, SUPER, and FarmCPU; for LFW, 3, 2, 2 and 27 were identified by GLM, MLM, SUPER and FarmCPU, and for LEAVES, 1, 2, 1, and 16 SNPs were identified by MLM, cMLM, SUPER, and FarmCPU. Of the SNPs described above, 36 were significantly associated with drought-tolerant traits repeatedly detected at least in two environments or two models (Table 3; Supplementary Figure 2).

**Table 3:** Significant SNPs associated with drought-tolerance traits that were repeatedly detected in at least two environments or two models.

**Traits**	**SNP**	**Chr**	**Position**	**GLM**		**MLM**		**CMLM**		**SUPER**		**FarmCPU**	
				**-logP**	**Env**.	**-logP**	**Env**.	**-logP**	**Env**.	**-logP**	**Env**.	**-logP**	**Env**.
CHL	TM5832	A3	621,671	4.98	E_1_	5.38	E_1_	5.15	E_1_	5.16	E_1_	11.39	E_1_
CHL	TM62605	D6	5,487,6807	4.82	E_1_	5.06	E_1_	4.84	E_1_	4.85	E_1_		
CHL	TM62606	D6	54,884,942	5.16	E_1_	5.06	E_1_	4.84	E_1_	4.85	E_1_		
CHL	TM62607	D6	54,890,311	4.98	E_1_	5.38	E_1_	5.18	E_1_	5.19	E_1_		
CHL	TM62609	D6	54,911,815	4.98	E_1_	5.20	E_1_	5.00	E_1_	5.00	E_1_		
CHL	TM62610	D6	54,916,052	4.86	E_1_	5.20	E_1_	5.00	E_1_	5.00	E_1_		
CHL	TM62611	D6	54,918,765	4.98	E_1_	5.09	E_1_	4.89	E_1_	4.89	E_1_		
CHL	TM62612	D6	54,926,607	5.21	E_1_	5.20	E_1_	5.00	E_1_	5.00	E_1_		
CHL	TM62613	D6	54,951,583	4.86	E_1_	5.43	E_1_	5.23	E_1_	5.24	E_1_		
CHL	TM62614	D6	54,954,140	5.16	E_1_	5.09	E_1_	4.89	E_1_	4.89	E_1_		
CHL	TM62617	D6	54,970,026	4.79	E_1_	5.38	E_1_	5.18	E_1_	5.19	E_1_		
CHL	TM62621	D6	54,996,965			5.10	E_1_	4.87	E_1_	4.89	E_1_	5.41	E_1_
CHL	TM62623	D6	55,009,088			4.97	E_1_	4.74	E_1_	4.75	E_1_		
CHL	TM62625	D6	55,026,490			4.97	E_1_	4.74	E_1_	4.75	E_1_		
CHL	TM62626	D6	55,042,409			4.97	E_1_	4.74	E_1_	4.75	E_1_		
CHL	TM69863	D8	64,527,910	4.82	E_1_	5.02	E_3_	4.78	E_3_	4.85	E_3_	7.64	E_3_
LA	TM57176	D5	9,970,466	4.80	BLUP					4.88	BLUP		
PH	TM31805	A9	43,528,559	5.34	E_1_	5.81	E_1_	5.68	E_1_	5.51	E_1_	5.46	E_1_
PH	TM76073	D11	19,273,291	4.93	E_1_	5.31	E_1_			5.07	E_1_	5.43	E_1_
PH	TM76072	D11	19,269,898	4.74	E_1_	5.13	E_1_			4.88	E_1_	5.29	E_1_
SD	TM46636	A13	60,446,545	4.98	E_4_	5.04	E_4_	4.72	E_4_	4.86	E_4_		
SD	TM74311	D10	28,156,527	4.89	E_4_	5.05	E_4_			4.87	E_4_		
SD	TM74312	D10	28,162,597	4.89	E_4_	5.05	E_4_			4.87	E_4_		
SD	TM74314	D10	28,181,084	4.76	E_4_							5.98	E_4_
LEAVES	TM10267	A5	5,328,342			5.00	BLUP	5.11	BLUP	4.98	BLUP	5.62	BLUP
LFW	TM18554	A7	2,232,287									4.80; 7.73	E_1_; E_3_
LFW	TM40383	A12	7,095,050	4.74	E_3_	4.72	E_3_			4.71	E_3_	4.98	E_3_
LFW	TM73079	D10	560,544	5.29	E_1_	5.37	E_1_			5.36	E_1_		
LWW	TM59672	D6	11,843,657					5.35	E_2_	4.87	E_2_	7.65	E_2_
LWW	TM59675	D6	11,881,468					5.38	E_2_	4.91	E_2_		
LWW	TM59677	D6	11,906,406					5.38	E_2_	4.91	E_2_		
LWW	TM73079	D10	560,544	4.73	E_1_	4.80	E_1_			4.84	E_1_		
LWW	TM73946	D10	12,123,707									6.05;6.43	E_1_; E_4_
LDW	TM33865	A10	2,761,881	5.21	E_3_	5.08	E_3_	5.02	E_3_	5.04	E_3_	6.51;9.80	E_4_; E_3_
LDW	TM47696	A13	79,134,335									5.26; 6.50	E_3_; E_4_
LDW	TM72359	D9	40,414,934	4.83	E_4_	4.88	E_4_	4.98	E_4_	4.98	E_4_	8.47	E_4_
LDW	TM72361	D9	40,432,377			4.73	E_4_	4.84	E_4_	4.84	E_4_		
LDW	TM73079	D10	560,544	5.26	E_1_	5.30	E_1_			5.29	E_1_	4.73	E_1_

*E_1_, E_2_, E_3_, and E_4_ indicate the four environments 2015–2016 Damao in the field, 2015-2016 Damao in the greenhouse, 2016–2017 Yacheng in the greenhouse, and 2016-2017 Baogang in the field, respectively. CHL, chlorophyll content; LA, leaf area; PH, plant height; SD, stem diameter; LEAVES, number of leaves; LFW, leaf fresh weight; LWW, leaf wilted weight; and LDW, leaf dry weight. GLM, general linear model; MLM, mixed linear model; CMLM, compressed linear mixed model; SUPER, settlement of kinship under progressively exclusive relationship; FarmCPU, fixed and random model circuitous probability unification*.

Among the 36 SNPs, 24 SNPs, which were respectively associated with 5 traits, formed 5 association peaks. For CHL, an association peak, including 14 significantly associated SNPs (TM62605, TM62606, TM62607, TM62609, TM62610, TM62611, TM62612, TM62613, TM62614, TM62617, TM62621, TM62623, TM62625, and TM62626), was detected in a 165.60 kb region on chromosome D6 and was significantly associated with the E_1_ values. For PH, two SNPs (TM76072 and TM76073) formed a GWAS peak, which was detected at a distance of 3.4 kb on chromosome D11. These two SNPs were significantly associated with the E_1_ values, simultaneously detected by GLM, MLM, SUPER, and FarmCPU. For SD, three SNPs (TM74311, TM74312, and TM74314) formed an association peak, which was detected at a distance of 24.56 kb on chromosome D10. They were significantly associated with the E_4_ values detected by GLM, MLM, SUPER, and FarmCPU. For LWW, an association peak, including 3 SNPs (TM59672, TM59675, and TM59677), was identified at a distance of 62.75 kb on chromosome D6. These SNPs were significantly associated with the E_2_ values detected by the MLM, CMLM, and FarmCPU models. For LDW, an association peak including 2 SNPs (TM72359 and TM72361), was detected at a distance of 17.44 kb on chromosome D9. These SNPs were significantly associated with the E_4_ values detected by the GLM, MLM, CMLM, SUPER, and/or FarmCPU models.

In addition, one SNP, TM73079, located on chromosome D10, was simultaneously associated with LFW, LWW, and LDW, which could result from the pleiotropy of a single causal gene or the tight linkage of multiple causal genes.

### Comparison of the Significantly Trait-Associated SNPs With Those From Previous Reports

**Figure 4:**
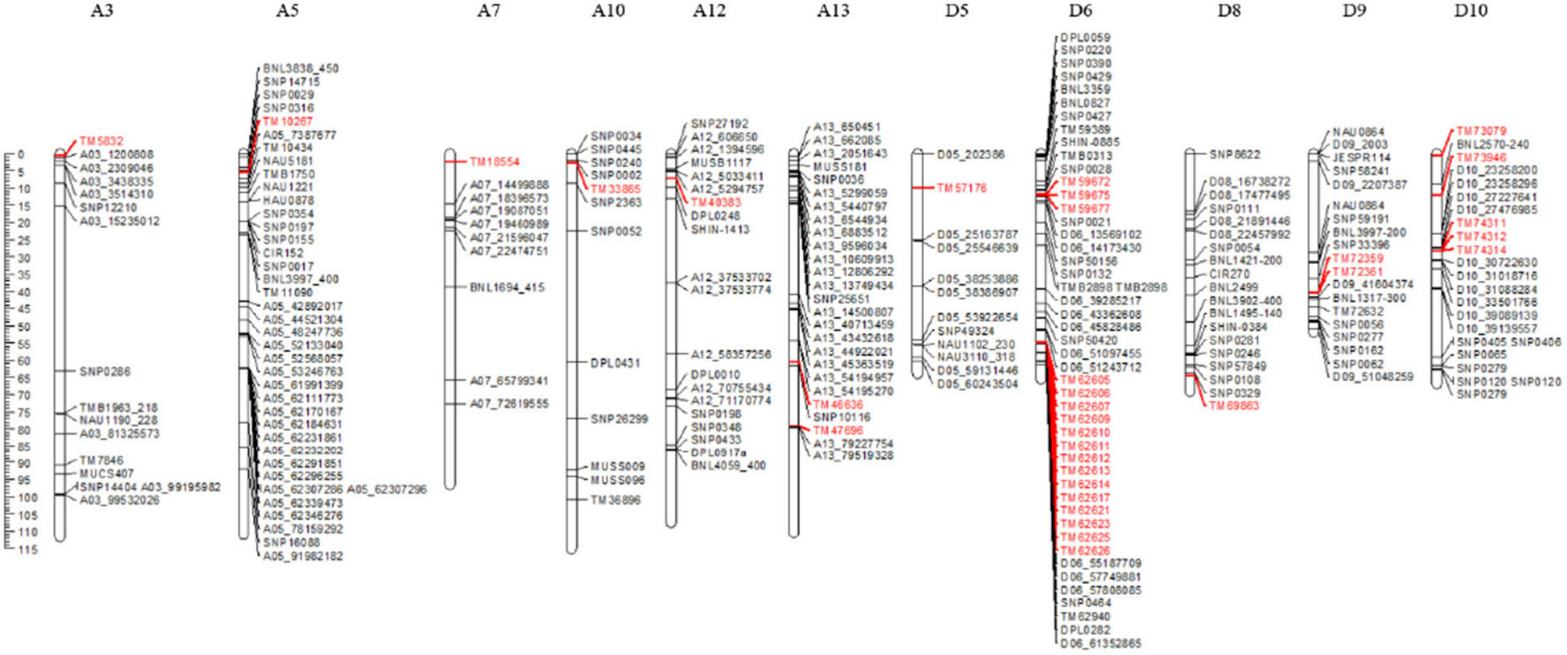
Meta-analysis of markers related to drought-tolerance traits. The markers shown in red represent the SNPs detected in this study.

In the current study, the 36 trait-associated SNPs detected repeatedly were located on chromosomes A3, A5, A7, A9, A10, A12, A13, D5, D6, D8, D9, D10, and D11. To validate these SNPs, we investigated previously mapped markers/QTL from 23 publications on drought tolerance in cotton, and obtained 426 markers located on these 11 chromosomes. The physical locations of these markers were searched by e-PCR. Finally, nine SNPs, TM47696, TM33865, TM40383, TM10267, TM59672, TM59675, TM59677, TM72359, and TM72361, which were identified in the current study and located on chromosomes A13, A10, A12, A5, D6, and D9, were localized within or near previously reported markers/QTL for drought tolerance (Figure 4). Specifically, the marker TM47696 on chromosome A13 associated with LDW was near *qDSW-A13-5* (A13_79227754—A13_79519328), which was detected by Abdelraheem et al. (2021b), with the distance between them being 90 kb. The marker TM33865 on chromosome A10 associated with LDW was near *qDSW.PEG3NM.c10* (SNP0445–SNP0002), which was identified by Abdelraheem et al. (2017), with the distance between them being 810 kb. The marker TM40383 on chromosome A12 associated with LFW was near *qtl_SLW_A12_E2-1* (A12_5033411 – A12_5294757), which was detected in our previous study (Magwanga et al., 2020), with the distance between them being 1.81 Mb. The marker TM10267 on chromosome A5 associated with the number of leaves was near a QTL (SNP0029–SNP0316) for LAI and *qtl_SLW_A05_CA-2* (A05_7387677–A05_91982182), which were detected by Pauli et al. (2016) and/or detected in our previous research (Magwanga et al., 2020), with the distances between them being 300 kb and 2.06 Mb, respectively. The three markers, TM59672, TM59675 and TM59677, which were located on chromosome D6 and associated with LWW, were on the QTL (SNP0132–SNP0028) of LAI overlap detected by Pauli et al. (2016), and were near the two SNPs (D06_13569102 and D06_14173430) associated with DSW, which were detected by Abdelraheem et al. (2021a), with the distances between them being 1.66 Mb and 2.26 Mb, respectively. The two markers, TM72359 and TM72361 on chromosome D9, were associated with LDW, located near SNP33396, which was associated with DSW by Mahmood et al. (2019), with the distance between them being approximately 4 Mb.

### Distribution and Annotation of Candidate Genes

According to previous studies (Sun Z. et al., 2017; Li et al., 2018; Song et al., 2019), the genes located within ± 200 kb of the 36 significantly associated SNPs were collected based on their position in the *G. hirsutum* genome in this study. According to this rule, a total of 520 genes were collected (Supplementary Table 4). We then compared the distributions of these genes between A and D subgenome. The results showed that the number of genes detected within the D subgenome (275) was not significantly different from that within the A subgenome (245). On the A subgenome, chromosome A13 contained the largest numbers of genes (61), whereas chromosome A9 had only five genes. On the D subgenome, chromosome D10 had the largest number of genes (86), whereas chromosome D11 had only 27 genes. In addition, we observed the position of these candidate genes and found that eight genes included one significantly associated SNP, respectively (Table 4).

**Table 4:** Candidate genes that include significantly associated SNPs for traits involved in drought tolerance.

**Gene ID**	**SNPs**	**Chr**.	**Position**	**Ref**	**Alt**	**Traits**	**Ortholog**	**Annotation**
Gh_A10G0304	TM33865	A10	2761881	C	A	LDW	AT5G45650.2	Subtilase family protein
Gh_A13G1963	TM47696	A13	79134335	A	C	LDW	AT2G29560.1	A putative phosphoenolpyruvate enolase
Gh_A05G0492	TM10267	A5	5328342	G	A	LEAVES	AT5G25080.1	Sas10/Utp3/C1D family
Gh_A07G0173	TM18554	A7	2232287	G	A	LFW	AT5G11520.1	The chloroplastic isozyme of aspartate aminotransferase
Gh_D05G1158	TM57176	D5	9970466	A	C	LA	AT1G15780.3	Mediator of RNA polymerase II transcription subunit 15a-like protein
Gh_D06G0687	TM59675	D6	11881468	A	G	LWW	AT4G27190.1	NB-ARC domain-containing disease resistance protein
Gh_D06G0689	TM59677	D6	11906406	G	A	LWW	AT5G11700.2	Ephrin type-B receptor
Gh_D08G2462	TM69863	D8	64527910	A	C	CHL	AT5G11270.1	A homeodomain transcription factor

*CHL, chlorophyll content; LA, leaf area; LEAVES, number of leaves; LFW, leaf fresh weight; LWW, leaf wilted weight; and LDW, leaf dry weight*.

To predict the functions of these 520 genes, each was annotated with GO terms. Three hundred and twenty-seven genes had annotation information, and were classified into three main types including molecular function, biological process, and cellular components (Supplementary Table 4; Supplementary Figure 3). In the molecular function category, most genes were enriched in binding, catalytic activity, transferase activity, kinase activity, oxidoreductase activity, transporter activity, and phosphatase activity. In the biological process category, 119 genes were related to stimulus response, such as water deprivation, desiccation, cadmium ion, salt stress, cold, light, wounding, bacterium, auxin, abscisic acid; 30 genes were involved in multiple signaling pathway, including ethylene, jasmonic acid, abscisic acid, auxin, and gibberellic acid mediated signaling pathway; 62, 55, and 39 genes were involved in transport, growth and developmental process, and regulation, respectively. In the cellular component category, 104 genes were mainly related to membrane and the integral component of the membrane.

### Transcriptome Sequencing Analysis

**Figure 5:**
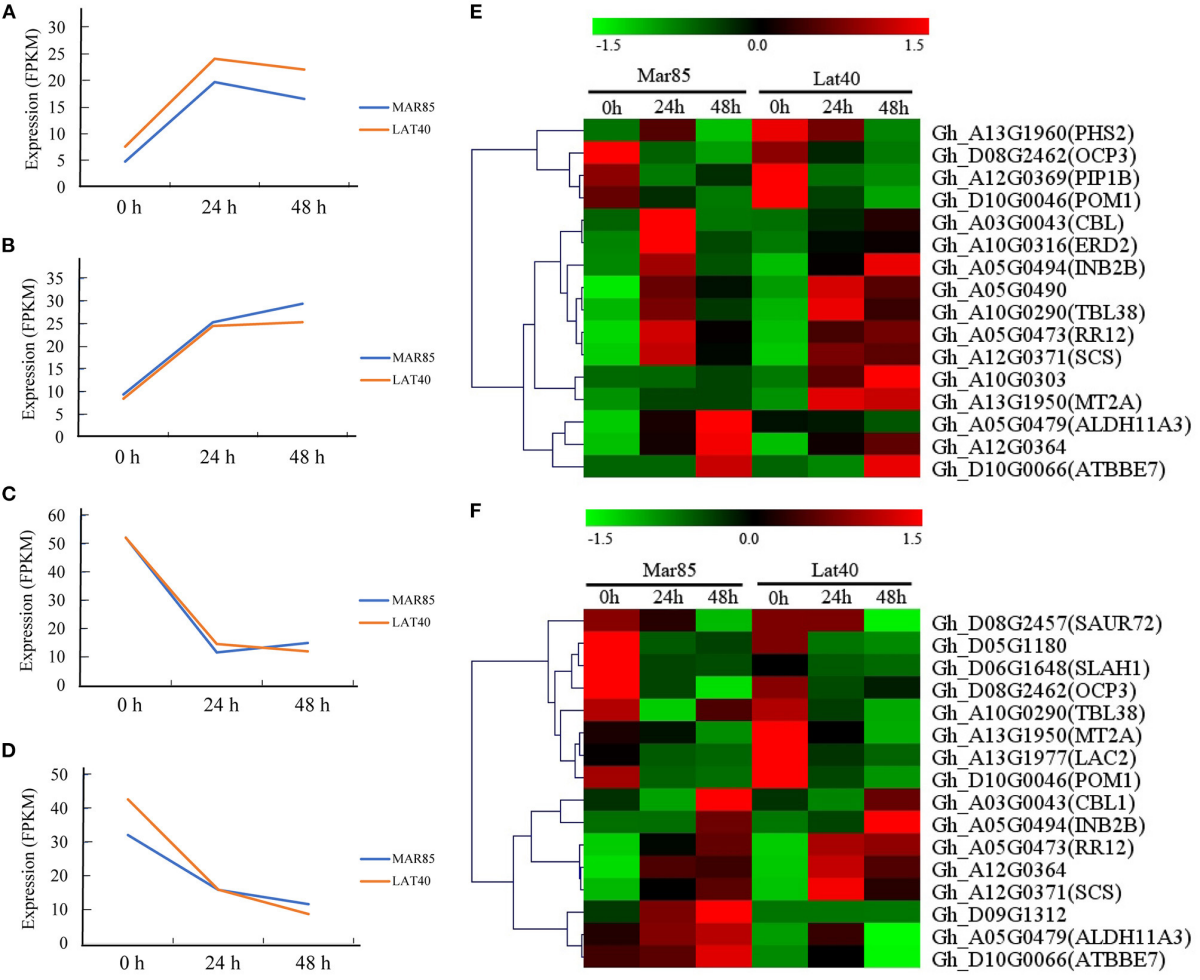
The expression change trends of selected genes. **(A)** Genes down regulated in leaf tissue. **(B)** Genes down regulated in root tissue. **(C)** Genes up regulated in leaf tissue. **(D)** Genes up regulated in root tissue. **(E)** expression pattern of drought-tolerance candidate genes in leaf tissue. **(F)** expression pattern of drought-tolerance candidate genes in root tissue.

To explore the response patterns of these 520 genes under drought, we carried out transcriptome sequencing using the leaf and root tissues of Mar85 and Lat40 at 0 h, 24 h and 48 h after 17% PEG6000 treatment. Among these genes, 278 and 256 genes, barely or weakly expressed (FPKM values <5) in leaf or root tissue, were removed. Moreover, the expression changes of another 43 and 49 genes in both tissues were <2-fold. The remaining 199 and 215 genes were divided into two categories in both tissues (Figures 5A–D; Supplementary Figure 4). After 24 h of drought treatment, 142 and 132 genes showed upregulation in leaf and root tissues, respectively (Figures 5A,B); while the expression of the other 57 and 83 genes displayed downregulated expression in these two tissues (Figures 5C,D).

Combined with the gene annotations of their homologs in Arabidopsis, 21 genes may be involved in the drought response (Figures 5E,F). Among these genes, the expression pattern of nine genes showed the same change trend in both leaf and roots, seven of which (*Gh_A03G0043, Gh_A05G0473, Gh_A05G0479, Gh_A05G0494, Gh_A12G0364, Gh_A12G0371*, and *Gh_D10G0066*, which are homologous to *AT4G17615.3, AT2G25180.1, AT2G24270.3, AT5G57910.1, AT2G21180.1, AT4G38810.2*, and *AT1G26420.1*, respectively) were upregulated, and two of which (*Gh_D08G2462* and *Gh_D10G0046*, which are homologous to *AT5G11270.1* and *AT1G05850.2*, respectively) were downregulated. Moreover, two genes (*Gh_A10G0290* and *Gh_A13G1950*, which are homologous to *AT1G29050.1* and *AT3G09390.2*, respectively) displayed opposite change trends, with decreased expression in root tissue and increased expression in leaf tissue. Of the remaining ten genes, three (*Gh_A05G0490, Gh_A10G0303*, and *Gh_A10G0316*, which are homologous to *AT4G31830.1, AT1G29640.1*, and *AT1G29330.1*) were increased in leaf tissue, one (*Gh_D09G1312*, which is homologous to *AT3G52710.1*) was increased in root tissue, two (*Gh_A12G0369* and *Gh_A13G1960*, which are homologous to *AT2G45960.1* and *AT3G46970.1*) was decreased in leaf tissue, and four (*Gh_A13G1977, Gh_D05G1180, Gh_D06G1648*, and *Gh_D08G2457*, which are homologous to *AT2G29130.1, AT3G15810.1, AT1G62280.1*, and *AT3G12830.1*, respectively) were decreased in the root tissue.

## Discussion

### Abundant Genetic Variation Among *G. hirsutum* Race Accessions Is the Basis for Performing a GWAS

SNPs have become the preferred and widely used marker system for analyzing the genetic diversity of biological species because of the potential to identify a substantial number of SNPs throughout the whole genome. PIC is an index for evaluating the allele frequencies at single loci or their sum over multiple loci, which reflects the genetic diversity of the population. Several studies have reported the PIC of *G. hirsutum* based on SNPs. Based on the Cotton 63K Illumina Infinium SNP array, Sun Z. et al. (2017) genotyped a natural population of 719 Upland cotton accessions, identified 10,511 polymorphic SNPs, and calculated the mean PIC of the A and D subgenome as 0.287 and 0.283, respectively; Song et al. (2019) genotyped 279 Upland cotton accessions and calculated the mean PIC of the whole genome as 0.250 with 10,660 high-quality polymorphic SNPs. Based on the Cotton80KSNP array, Xing et al. (2019) evaluated the mean PIC of 196 Upland cotton accessions as 0.267 with 41,815 polymorphic SNPs. Using 169 cultivated Upland cotton accessions, Li et al. (2018) identified the mean PIC values of the A and D subgenome as 0.282 and 0.281, respectively, with 49,650 polymorphic SNPs. Here, we identified the mean PIC of 189 accessions of *G. hirsutum* as 0.3273 using 51,268 polymorphic SNPs. The results above suggest that the diversity of *G. hirsutum* races is greater than that of cultivated Upland cotton, which was consistent with previous reports (Liu et al., 2000; Lacape et al., 2006; Abdurakhmonov et al., 2008; Hinze et al., 2015, 2016, 2017; Wang et al., 2016; Okubazghi et al., 2017). The complex natural habitat and changeable climate where *G. hirsutum* races originated, which lacked strong selection pressure, played a decisive role in the accumulation of genetic variation. In contrast, Hou et al. (2018) genotyped 319 cultivars using the Cotton80KSNP array, produced 55,060 polymorphic SNPs, and calculated the mean PIC of the A and D subgenome as 0.338 and 0.334, respectively. Su et al. (2018) genotyped 355 cultivated *G. hirsutum* accessions through specific-locus amplified fragment sequencing, detected 93,250 polymorphic SNPs, and calculated the mean PIC as 0.34. It is undeniable that domestication can create some diversity. However, the diversity analyses are characteristically limited by the number of markers and the number of materials used. Therefore, many more accessions of *G. hirsutum* races will be used to evaluate the genetic diversity efficiently through genotyping by sequencing, which produces many more polymorphic SNPs, and that genetic diversity can be fully utilized in current breeding programs.

### The Character of Drought-Tolerant Genetic Variation

The drought tolerance of cotton is a complex and comprehensive quantitative trait. In this study, the *H*^2^ of eight drought relative traits meant that 30% to 60% of the phenotypic variation of these traits is determined by genetic factors. Similarly, Pauli et al. (2016), Zheng et al. (2016), Abdelraheem et al. (2017, 2021a), and Li B. Q. et al. (2020) estimated the *H*^2^ for DT were 0.12–0.88, 0.40–0.666, 0.23–0.60, 0.39–0.65, and 0.08–0.76. On the contrary, Abdelraheem et al. (2015a, 2021b) estimated the *H*^2^ for DT were 0.60–0.75, and 0.681–0.691 under drought stress. The results above indicated that drought tolerance was heavily influenced by environments, and that QTL identification was urgently needed for excavating the accumulation of minor genes and major genes on DT.

In this study, based on a *G. hirsutum* races population with abundant genetic variation, we carried out a GWAS of eight drought-tolerant traits and identified eight and twenty-eight significantly associated SNPs in the A subgenome and D subgenome, respectively. Similarly, several previous publications indicated that more QTL for drought stresses were detected in the D subgenome than those in the A subgenome (Jia et al., 2014; Abdelraheem et al., 2015a,b, 2017, 2019, 2021a; Hou et al., 2018). Similarly, several researchers reported that the number of QTL for salt stress in the D subgenome was greater than that in the A subgenome (Du et al., 2016; Oluoch et al., 2016; Yasir et al., 2019; Yuan et al., 2019). The results above supported that the D subgenome contributed to adaptation during the long-term domestication of allotetraploid *G. hirsutum* (Zhang et al., 2015). However, in our previous research, the QTL for drought stress were mapped asymmetrically within the two subgenomes, including 17 and 13 QTL located in the A and D subgenomes, respectively (Magwanga et al., 2020). Similarly, Abdelraheem et al. (2021b) detected 13 and 8 QTL for DT in the A and D subgenome, respectively. The contribution of the D subgenome to drought tolerance needs to be more widely investigated based on more indicators and populations with greater genetic diversity.

Among all 36 significantly associated SNPs identified in the present study, TM73079 on chromosome D10 was simultaneously associated with LFW, LWW, and LDW. Moreover, phenotypic analysis showed that these three traits were significantly highly correlated with each other. These results may be due to the pleiotropy of a single causal gene or the tight linkage of multiple causal genes. Furthermore, another nine SNPs, TM47696, TM33865, TM40383, TM10267, TM59672, TM59675, TM59677, TM72359, and TM72361, located on chromosomes A13, A10, A12, A5, D6, and D9, were localized within or near previously reported markers/QTL for drought tolerance (Figure 4). Four SNPs, TM47696, TM33865, TM72359, and TM72361, associated with LDW were localized within QTL detected for DSW (Abdelraheem et al., 2017, 2021b); three SNPs, TM59672, TM59675, and TM59677, associated with LWW, overlapped with a QTL for LAI (Pauli et al., 2016), and were near D06_13569102 and D06_14173430, which were detected associated with DSW (Abdelraheem et al., 2021b); TM40383, associated with LFW, was near a QTL for SLW (Magwanga et al., 2020); and TM10267, associated with LEAVES, was near QTL for LAI and SLW (Pauli et al., 2016; Magwanga et al., 2020). Similarly, Li et al. (2019) detected a QTL on A11 at 86.28 Mb that associated with the plant wilting score under drought stress; this QTL overlapped with the QTL for PH at 74.64–89.93 Mb detected by (Abdelraheem et al., 2021a). The colocalized QTL found for different traits suggest that there may be a common response to drought stress and these regions may be involved in drought tolerance. Moreover, moderately or highly significant correlations (p <0.01) among LDW, LWW, LFW, LA, and LEAVES were observed in the present study, indicating that these traits have a close relationship with each other, and suggesting that they are better indicators for analyzing the genetic basis of drought tolerance in *G. hirsutum*.

Moreover, we compared the genomic positions of the 36 significantly associated SNPs with the relative candidate genes and subsequently identified that only eight significant SNPs were distributed within separate putative candidate genes. However, transcriptome sequencing could provide expression level of each gene and the variations in the gene coding regions. Using transcriptome-wide association studies (TWAS) to associate the target trait and the expression level of genes in the whole genome is available for predicting candidate genes in studying complex traits. Till now, TWAS has been successfully implemented in cotton and rapeseed to identify casual genes for important traits (Li Z. H. et al., 2020; Ma et al., 2021; Tang et al., 2021). For drought tolerance of *G. hirsutum* races, a transcriptome-wide association study (TWAS) should be confirmed to identify pivotal expression-trait association and to detect functional SNPs directly from transcriptionally active regions of the genome and potential genes.

### Potential Candidate Genes of Drought Tolerance

Using the *G. hirsutum* population containing 189 accessions, we identified 36 significant SNPs associated with eight drought-tolerance traits, analyzed the annotation and expression pattern of 520 candidate genes, and predicted that some genes may be involved in the drought response. For example, *Gh_D08G2462*, containing one significantly associated SNP, TM69863 (Table 4), is homologous to *AT5G11270.1* (OCP3, a member of the homeobox transcription factor family). The loss-of-function *Atocp3* mutant exhibits drought tolerance through the modulation of ABA-mediated stomatal closure (Ramirez et al., 2009). In addition, *Gh_A03G0043* is homologous to *AT4G17615.3* (CBL1), which codes for the calcineurin B-like (CBL) protein; this protein is a primary calcium sensor and plays crucial roles in the response to drought in plants (Cheong et al., 2003). Here the gene expression of *Gh_A03G0043* increased in leaf and root tissues after drought treatment, which is similar to our previous report on wild type Arabidopsis (Lu et al., 2019; Magwanga et al., 2019), and the expression of its homologous genes are similar in several plants, such as Arabidopsis (Cheong et al., 2003), *P. betulifolia* Bunge (Xu et al., 2015), and *C. sinensis* (Shu et al., 2020). Moreover, several reports indicated that overexpression of *CBL1* may confer tolerance to drought (Cheong et al., 2003). Another candidate gene, *Gh_A12G0369*, is an ortholog of *AT2G45960.1* (plasma membrane intrinsic protein 1, PIP1B), which encodes a plasma membrane intrinsic protein and functions as a water channel in plants. The gene expression of *Gh_A12G0369* decreased in both tissues after drought treatment in this study, which is consistent with the results in *A. thaliana* (Jang et al., 2004), *F. arundinacea* (Pawlowicz et al., 2017), and *S. bigelovii* (Sun X. et al., 2017). Many studies have indicated that PIP1B was involved in drought stress. For example, overexpression of *SbPIP1* in tobacco plants improved drought tolerance (Sun X. et al., 2017); overexpression of *NnPIP1-2, ScPIP1*, and *SpPIP1*, enhanced the tolerance of Arabidopsis to stresses (Chen et al., 2018; Wang et al., 2019; Liu et al., 2020). Overexpression of *AtPIP1;2* in transgenic tobacco plants increased plant vigor under favorable growth conditions but had no beneficial effects under drought stress (Aharon et al., 2003). However, the decrease of *PIP1b* gene expression in Arabidopsis resulted in reduced water permeability in leaf mesophyll protoplasts and an increased root:shoot ratio (Kaldenhoff et al., 1998). Meanwhile, *Gh_A05G0479* is homologous to *AT2G24270.3* (aldehyde dehydrogenase, *ALDH11A3*), *Gh_A13G1950* is homologous to *AT3G09390.2* (metallothionein 2a, *MT2A*), and *Gh_A05G0473* is homologous to *AT2G25180.1* (Response Regulators 12, *RR12*). These homologous genes in Arabidopsis may be involved in the drought response. These results suggest that *Gh_D08G2462, Gh_A03G0043*, and *Gh_A12G0369* may be candidate genes involved in the drought response, and future research will be directed to understanding their regulatory mechanisms.

## Data Availability Statement

The original contributions presented in the study are included in the article/Supplementary Materials, further inquiries can be directed to the corresponding author/s.

## Author Contributions

XC and YX designed the experiment. XG, YuaW, YH, RS, TQ, and ZH analyzed the data. XG, YH, ZZ, KW, FL, YuhW, XC, and YX carried out the experiments. XG, YuaW, and YH prepared the manuscript. All authors have read, discussed, and approved the current version of the manuscript.

## Funding

This work was supported by the Science and Technology Development Project of Henan Province (212102110054 and 222102110146), the Key Scientific Research Projects of Higher Education of Henan Province (22A210014), State Key Laboratory of Cotton Biology Open Fund (CB2021A02), and the Postgraduate Education Reform and Quality Improvement Project of Henan Province (Yu degree [2018] No. 23).

## Conflict of Interest

The authors declare that the research was conducted in the absence of any commercial or financial relationships that could be construed as a potential conflict of interest.

## Publisher's Note

All claims expressed in this article are solely those of the authors and do not necessarily represent those of their affiliated organizations, or those of the publisher, the editors and the reviewers. Any product that may be evaluated in this article, or claim that may be made by its manufacturer, is not guaranteed or endorsed by the publisher.

## Supplementary Material

Supplementary Figure 1 Histogram of drought-tolerance traits investigated in four environments. E_1_, E_2_, E_3_, and E_4_ indicate the four environments 2015–2016 Damao in the field, 2015–2016 Damao in the greenhouse, 2016–2017 Yacheng in the greenhouse, and 2016–17 Baogang in the field, respectively; CHL, chlorophyll content; LA, leaf area; PH, plant height; SD, stem diameter; LEAVES, number of leaves; LFW, leaf fresh weight; LWW, leaf wilted weight; LDW, leaf dry weight.

Supplementary Figure 2 Manhattan plot of genome-wide association (GWA) mapping results for drought-tolerance traits in *G. hirsutum* races. 1–26 on the horizontal axis represent chromosome A1-A13, D2, D1, D7, D3, D13, D5, D10, D11, D4, D9, D8, D6, and D12, respectively; **(A)** Chlorophyll content (CHL); **(B)** Leaf area (LA); **(C)** Plant height (PH); **(D)** Stem diameter (SD); **(E)** Number of leaves (LEAVES); **(F)** Leaf fresh weight (LFW); **(G)** Leaf wilted weight (LWW); **(H)** Leaf dry weight (LDW); the dashed line represents the significance threshold (-log_10_*P* > 4.71).

Supplementary Figure 3 GO annotation of the genes within 200 kb upstream and downstream of the significantly associated SNPs.

Supplementary Figure 4 Heatmap of the expression levels of 199 and 215 genes in leaf and root tissues of MAR85 and LAT40 after drought treatment. **(A)** The 142 genes showed up-regulation in leaf tissue; **(B)** The 132 genes showed up-regulation in root tissue; **(C)** The 57 genes showed down-regulation in leaf tissue; **(D)** The 83 genes showed down-regulation in root tissue. Red indicates high expression, and green indicates low expression.

Supplementary Table 1 List of *G. hirsutum* accessions used in this study.

Supplementary Table 2 Data acquisition for eight traits in four environments. “Y” represented that data were collected; “-“ indicated that data were missing.

Supplementary Table 3 SNPs significantly associated with drought-tolerance that were detected using five models with GAPIT software. E_1_, E_2_, E_3_, and E_4_ indicate the four environments 2015–2016 Damao in the field, 2015–2016 Damao in the greenhouse, 2016–2017 Yacheng in the greenhouse, and 2016–2017 Baogang in the field, respectively. CHL, chlorophyll content; LA, leaf area; PH, plant height; SD, stem diameter; LEAVES, number of leaves; LFW, leaf fresh weight; LWW, leaf wilted weight; and LDW, leaf dry weight. GLM, general linear model; MLM, mixed linear model; CMLM, compressed linear mixed model; SUPER, settlement of kinship under progressively exclusive relationship; FarmCPU, fixed and random model circuitous probability unification.

Supplementary Table 4 Annotation of 520 genes identified based on 36 significantly associated SNPs.
