# Supplementary figures and images for: Genome-Wide Dissection of the Genetic Basis for Drought Tolerance in Gossypium hirsutum L. Races
Source: Front Plant Sci. 2022 Jun 28;13:876095. doi: 10.3389/fpls.2022.876095 (PMC9274165; doi:10.3389/fpls.2022.876095)

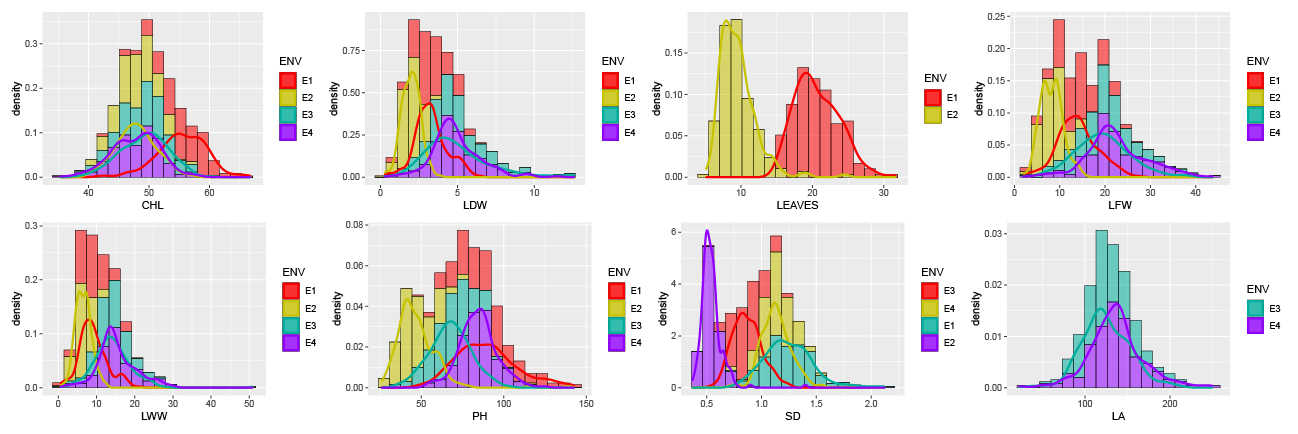

Supplement: Supplementary Figure 1 — Histogram of drought-tolerance traits investigated in four environments. E1, E2, E3, and E4 indicate the four environments 2015–2016 Damao in the field, 2015–2016 Damao in the greenhouse, 2016–2017 Yacheng in the greenhouse, and 2016–17 Baogang in the field, respectively; CHL, chlorophyll content; LA, leaf area; PH, plant height; SD, stem diameter; LEAVES, number of leaves; LFW, leaf fresh weight; LWW, leaf wilted weight; LDW, leaf dry weight. [file Data_Sheet_1.zip › Figure S1.TIF]

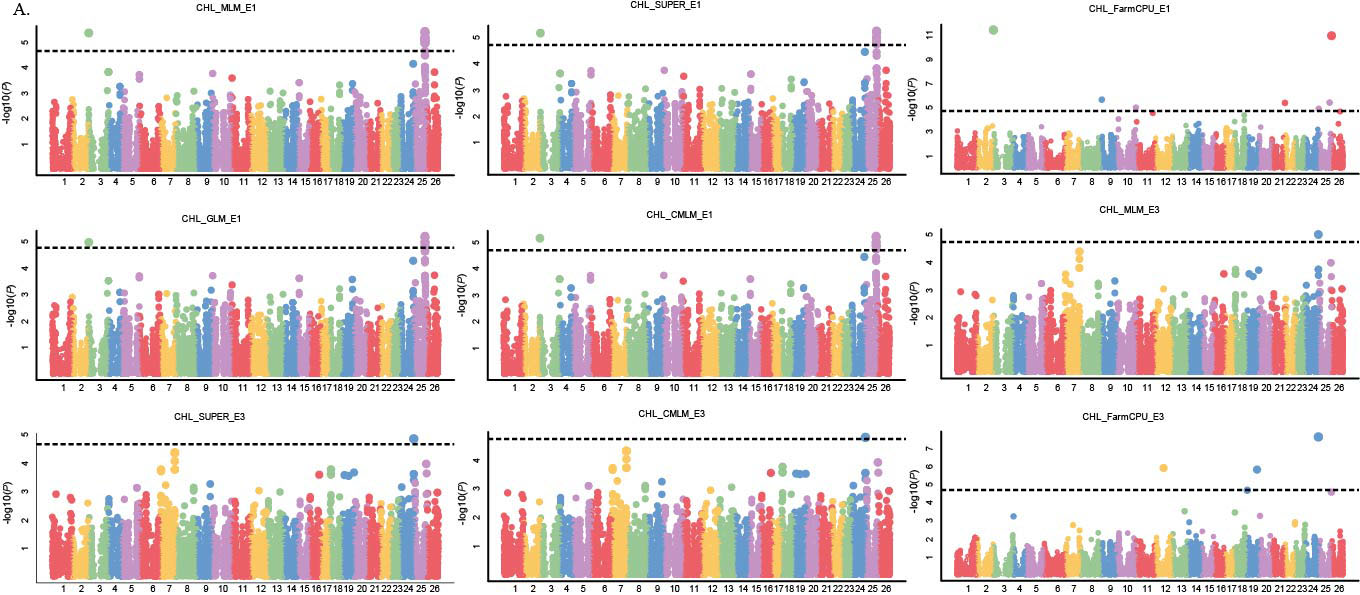

Supplement: Supplementary Figure 1 — Histogram of drought-tolerance traits investigated in four environments. E1, E2, E3, and E4 indicate the four environments 2015–2016 Damao in the field, 2015–2016 Damao in the greenhouse, 2016–2017 Yacheng in the greenhouse, and 2016–17 Baogang in the field, respectively; CHL, chlorophyll content; LA, leaf area; PH, plant height; SD, stem diameter; LEAVES, number of leaves; LFW, leaf fresh weight; LWW, leaf wilted weight; LDW, leaf dry weight. [file Data_Sheet_1.zip › Figure S2A.TIF]

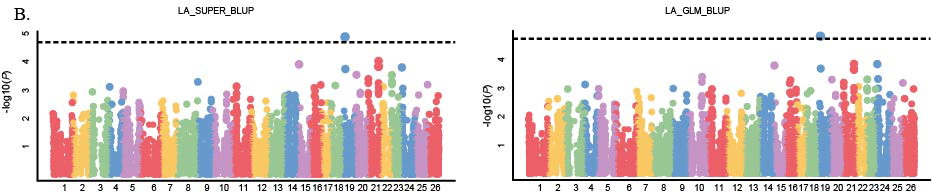

Supplement: Supplementary Figure 1 — Histogram of drought-tolerance traits investigated in four environments. E1, E2, E3, and E4 indicate the four environments 2015–2016 Damao in the field, 2015–2016 Damao in the greenhouse, 2016–2017 Yacheng in the greenhouse, and 2016–17 Baogang in the field, respectively; CHL, chlorophyll content; LA, leaf area; PH, plant height; SD, stem diameter; LEAVES, number of leaves; LFW, leaf fresh weight; LWW, leaf wilted weight; LDW, leaf dry weight. [file Data_Sheet_1.zip › Figure S2B.TIF]

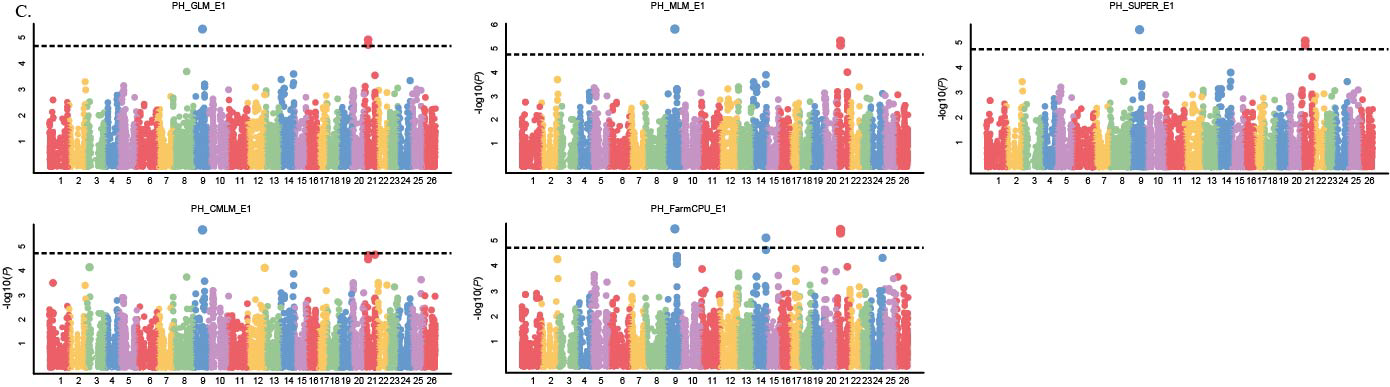

Supplement: Supplementary Figure 1 — Histogram of drought-tolerance traits investigated in four environments. E1, E2, E3, and E4 indicate the four environments 2015–2016 Damao in the field, 2015–2016 Damao in the greenhouse, 2016–2017 Yacheng in the greenhouse, and 2016–17 Baogang in the field, respectively; CHL, chlorophyll content; LA, leaf area; PH, plant height; SD, stem diameter; LEAVES, number of leaves; LFW, leaf fresh weight; LWW, leaf wilted weight; LDW, leaf dry weight. [file Data_Sheet_1.zip › Figure S2C.TIF]

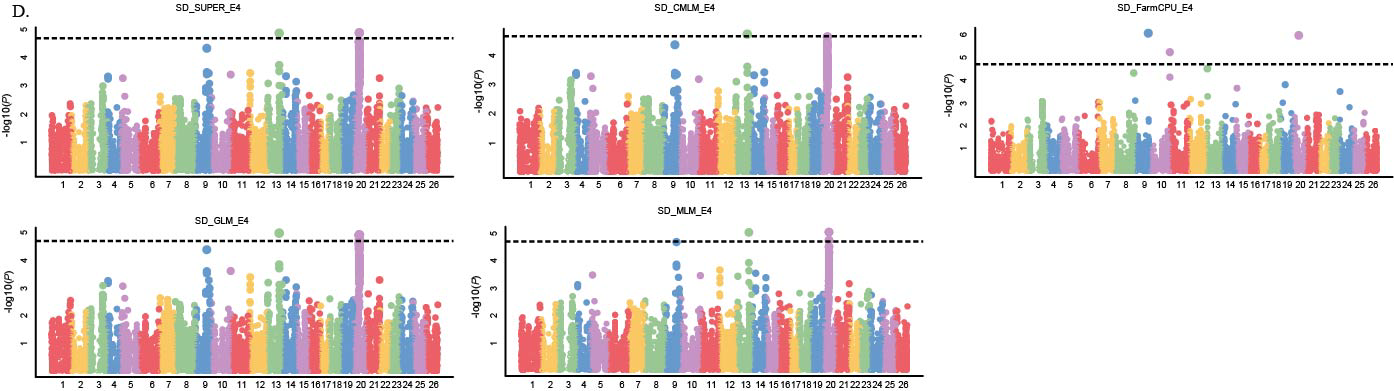

Supplement: Supplementary Figure 1 — Histogram of drought-tolerance traits investigated in four environments. E1, E2, E3, and E4 indicate the four environments 2015–2016 Damao in the field, 2015–2016 Damao in the greenhouse, 2016–2017 Yacheng in the greenhouse, and 2016–17 Baogang in the field, respectively; CHL, chlorophyll content; LA, leaf area; PH, plant height; SD, stem diameter; LEAVES, number of leaves; LFW, leaf fresh weight; LWW, leaf wilted weight; LDW, leaf dry weight. [file Data_Sheet_1.zip › Figure S2D.TIF]

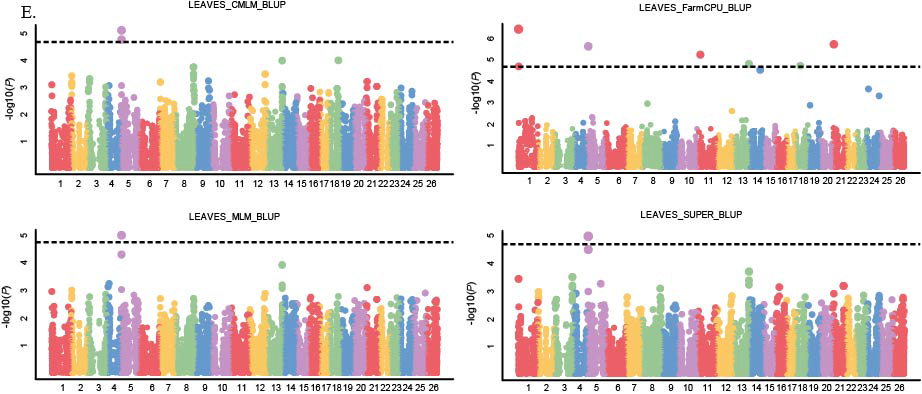

Supplement: Supplementary Figure 1 — Histogram of drought-tolerance traits investigated in four environments. E1, E2, E3, and E4 indicate the four environments 2015–2016 Damao in the field, 2015–2016 Damao in the greenhouse, 2016–2017 Yacheng in the greenhouse, and 2016–17 Baogang in the field, respectively; CHL, chlorophyll content; LA, leaf area; PH, plant height; SD, stem diameter; LEAVES, number of leaves; LFW, leaf fresh weight; LWW, leaf wilted weight; LDW, leaf dry weight. [file Data_Sheet_1.zip › Figure S2E.TIF]

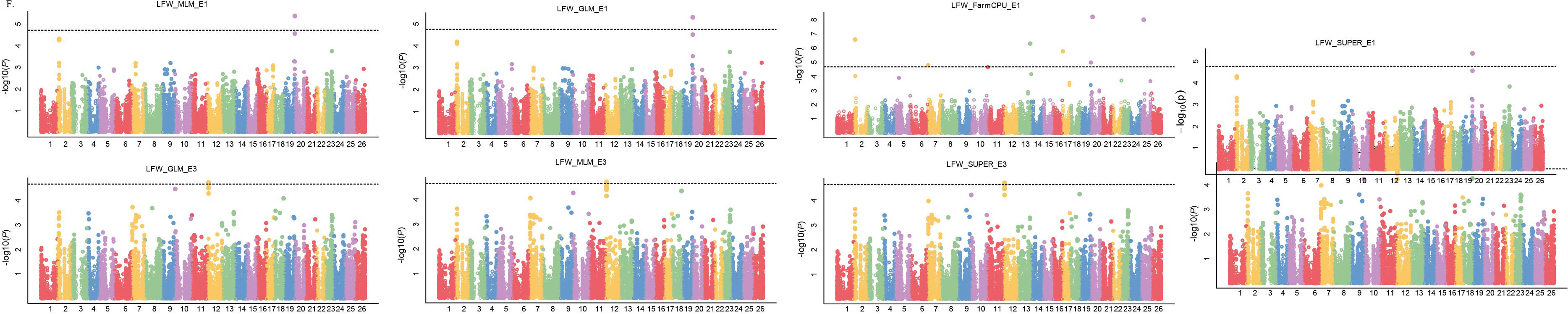

Supplement: Supplementary Figure 1 — Histogram of drought-tolerance traits investigated in four environments. E1, E2, E3, and E4 indicate the four environments 2015–2016 Damao in the field, 2015–2016 Damao in the greenhouse, 2016–2017 Yacheng in the greenhouse, and 2016–17 Baogang in the field, respectively; CHL, chlorophyll content; LA, leaf area; PH, plant height; SD, stem diameter; LEAVES, number of leaves; LFW, leaf fresh weight; LWW, leaf wilted weight; LDW, leaf dry weight. [file Data_Sheet_1.zip › Figure S2F.TIF]

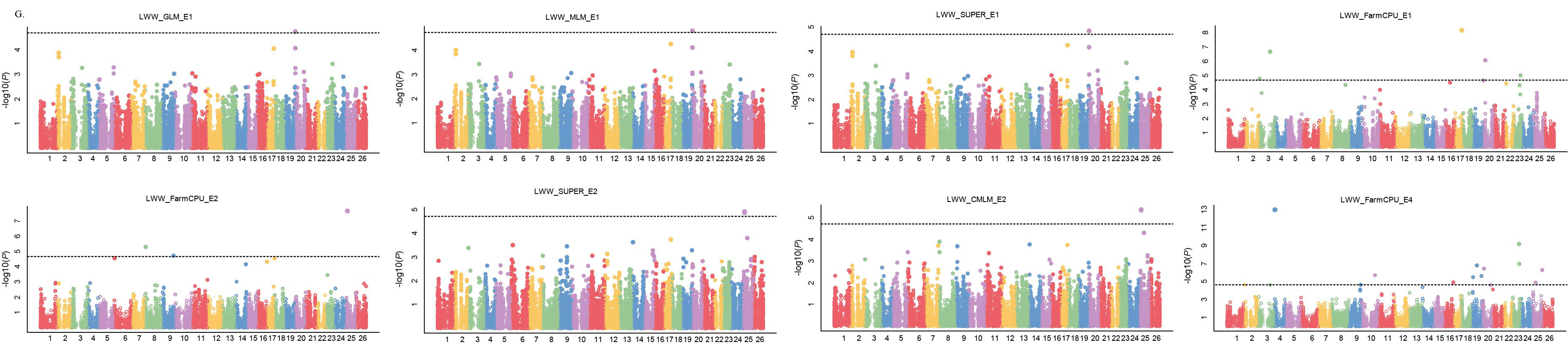

Supplement: Supplementary Figure 1 — Histogram of drought-tolerance traits investigated in four environments. E1, E2, E3, and E4 indicate the four environments 2015–2016 Damao in the field, 2015–2016 Damao in the greenhouse, 2016–2017 Yacheng in the greenhouse, and 2016–17 Baogang in the field, respectively; CHL, chlorophyll content; LA, leaf area; PH, plant height; SD, stem diameter; LEAVES, number of leaves; LFW, leaf fresh weight; LWW, leaf wilted weight; LDW, leaf dry weight. [file Data_Sheet_1.zip › Figure S2G.TIF]

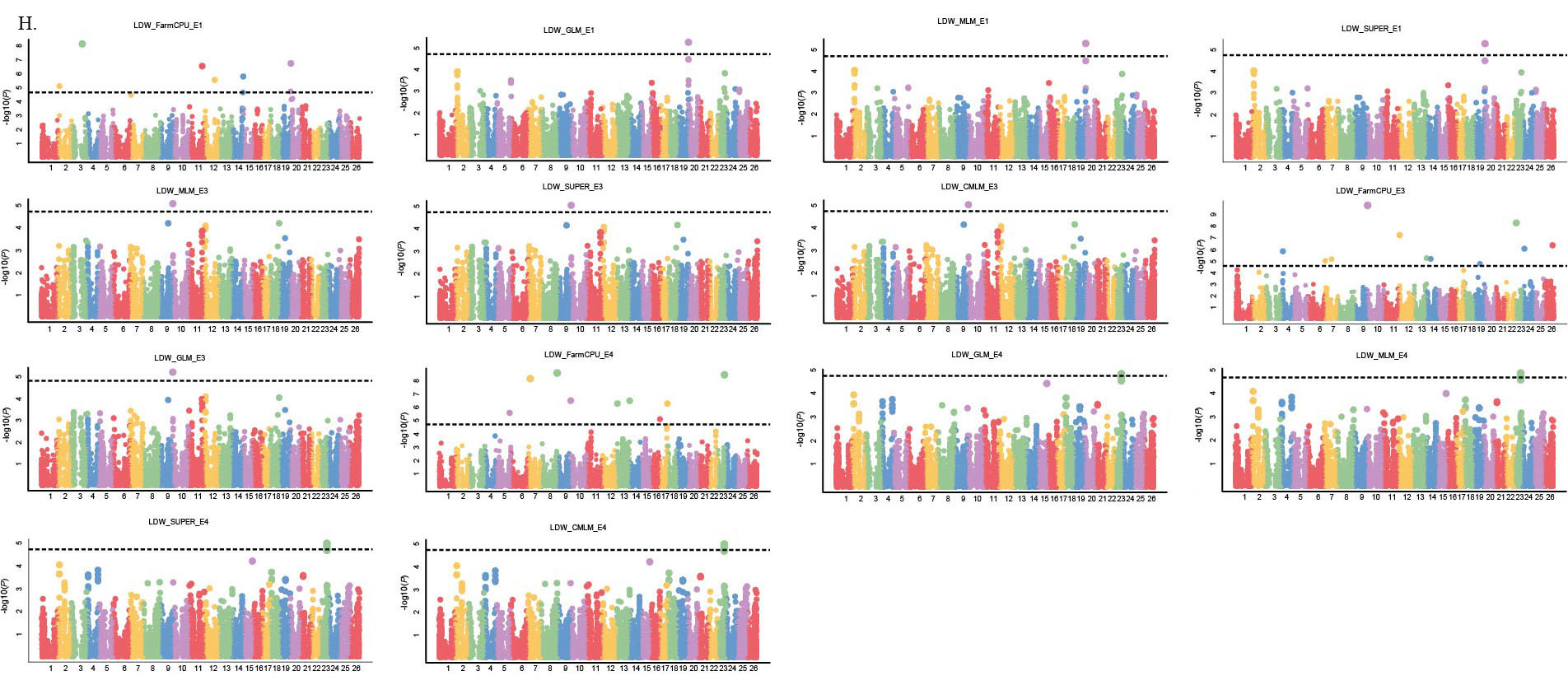

Supplement: Supplementary Figure 1 — Histogram of drought-tolerance traits investigated in four environments. E1, E2, E3, and E4 indicate the four environments 2015–2016 Damao in the field, 2015–2016 Damao in the greenhouse, 2016–2017 Yacheng in the greenhouse, and 2016–17 Baogang in the field, respectively; CHL, chlorophyll content; LA, leaf area; PH, plant height; SD, stem diameter; LEAVES, number of leaves; LFW, leaf fresh weight; LWW, leaf wilted weight; LDW, leaf dry weight. [file Data_Sheet_1.zip › Figure S2H.TIF]

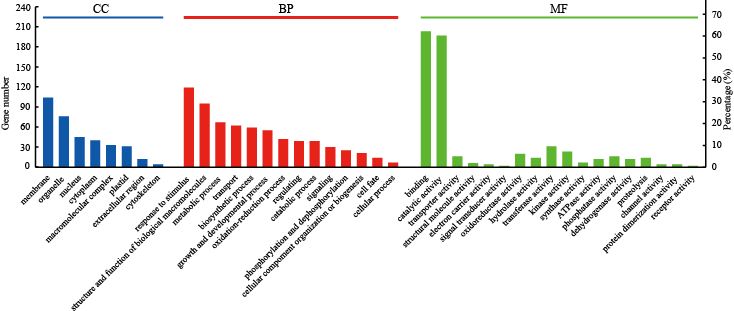

Supplement: Supplementary Figure 1 — Histogram of drought-tolerance traits investigated in four environments. E1, E2, E3, and E4 indicate the four environments 2015–2016 Damao in the field, 2015–2016 Damao in the greenhouse, 2016–2017 Yacheng in the greenhouse, and 2016–17 Baogang in the field, respectively; CHL, chlorophyll content; LA, leaf area; PH, plant height; SD, stem diameter; LEAVES, number of leaves; LFW, leaf fresh weight; LWW, leaf wilted weight; LDW, leaf dry weight. [file Data_Sheet_1.zip › Figure S3.TIF]

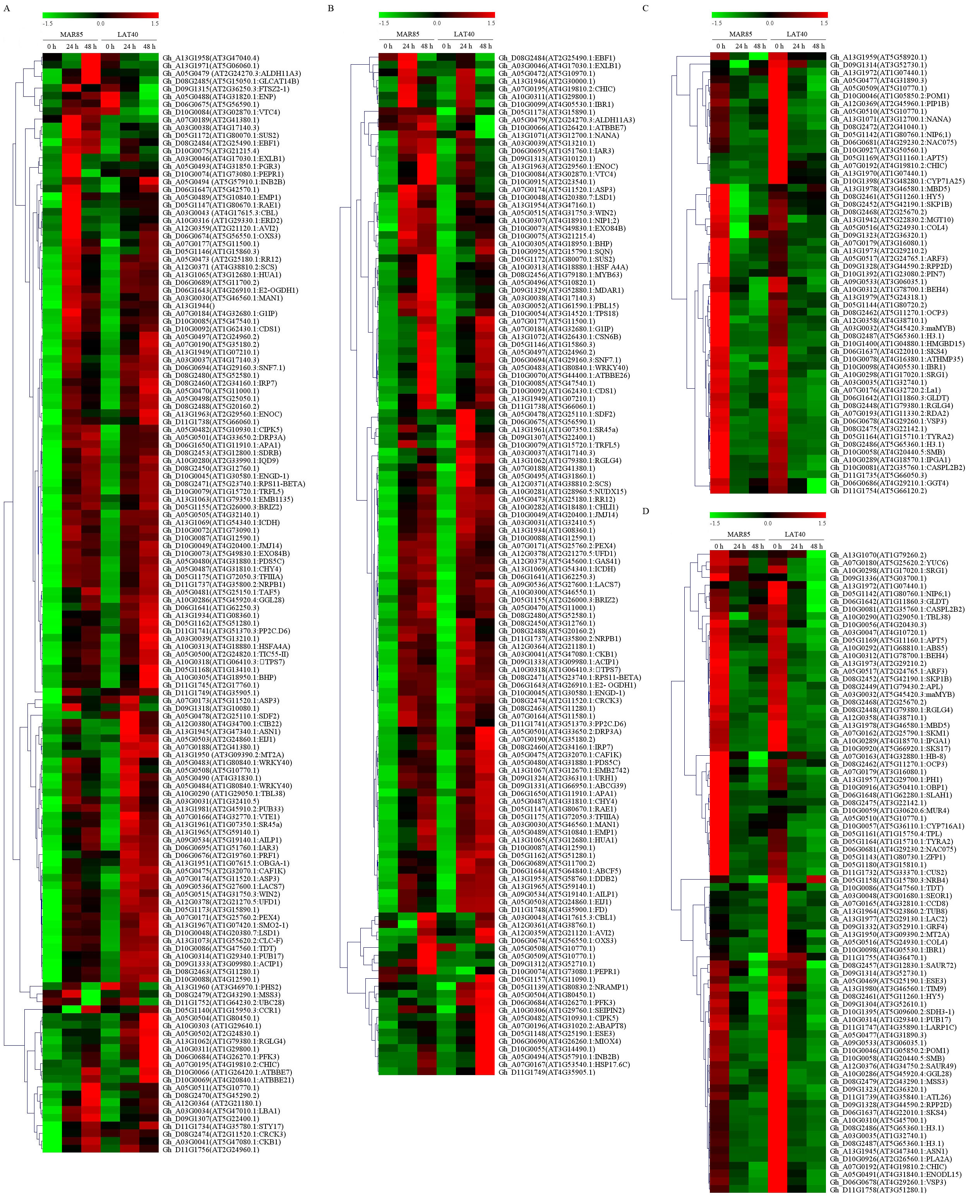

Supplement: Supplementary Figure 1 — Histogram of drought-tolerance traits investigated in four environments. E1, E2, E3, and E4 indicate the four environments 2015–2016 Damao in the field, 2015–2016 Damao in the greenhouse, 2016–2017 Yacheng in the greenhouse, and 2016–17 Baogang in the field, respectively; CHL, chlorophyll content; LA, leaf area; PH, plant height; SD, stem diameter; LEAVES, number of leaves; LFW, leaf fresh weight; LWW, leaf wilted weight; LDW, leaf dry weight. [file Data_Sheet_1.zip › Figure S4.TIF]
